# Progress and Challenges in the Electrocatalytic Reduction of Nitrate to Ammonia

**DOI:** 10.3390/molecules30193910

**Published:** 2025-09-28

**Authors:** Shupeng Yin, Yinglong Wang

**Affiliations:** 1 Shandong Vocational College of Science and Technology, Weifang 261053, China; 2 College of Chemical Engineering, Qingdao University of Science and Technology, Qingdao 266042, China

**Keywords:** nitrate, electrocatalytic nitrate reduction mechanism, catalyst design strategies

## Abstract

The escalating problem of nitrate pollution, coupled with the environmental burden of the Haber-Bosch process, has spurred intense interest in the electrocatalytic nitrate reduction reaction (eNO_3_RR) as a sustainable route for simultaneous wastewater treatment and ammonia production. However, the efficiency and selectivity of eNO_3_RR are hampered by the multi-step proton-coupled electron transfer process and the competing hydrogen evolution reaction. This review provides a comprehensive and critical overview of recent advances in understanding and designing catalysts for eNO_3_RR. We begin by elucidating the fundamental mechanisms and key reaction pathways, followed by a discussion on how critical parameters (e.g., electrolyte microenvironment, applied potential, reactor design) dictate performance. Further discussion of recent advances in catalysts, including single-metal catalysts, alloy catalysts, transition metal compounds, single-atom catalysts, carbon-based non-metal catalysts, and composite catalysts, highlights their significant roles in enhancing both the efficiency and selectivity. A distinctive feature of this review is its consistent critical assessment of catalysts through the dual lenses of practicality and sustainable development. Finally, we outline prevailing challenges and propose future research directions aimed at developing scalable and commercially viable electrocatalytic systems for green nitrogen management.

## 1. Introduction

Nitrate (NO_3_^−^) pollution in aquatic ecosystems has become a pressing global environmental issue, primarily driven by the extensive use of nitrogen-based fertilizers, fossil fuel combustion, and the discharge of industrial and domestic wastewater [1,2,3,4]. This widespread contamination disrupts the natural nitrogen cycle, leading to severe ecological consequences such as eutrophication, algal blooms, and subsequent hypoxia that threatens aquatic life [5,6]. Furthermore, NO_3_^−^ poses significant public health risks. Upon ingestion via contaminated drinking water or the food chain, it can be reduced to nitrite (NO_2_^−^), which can induce methemoglobinemia and form carcinogenic N-nitroso compounds, increasing the risks of various cancers and other health disorders [7,8]. In response, regulatory bodies worldwide have established stringent limits for NO_3_^−^ concentrations in water bodies, underscoring the urgency for effective remediation technologies [9].

Conventional technologies for NO_3_^−^ removal, such as ion exchange, reverse osmosis, and electrodialysis, primarily rely on physical separation [10,11,12]. While operationally simple, these methods often generate concentrated waste streams that require further treatment, posing risks of secondary pollution and increasing overall costs [13]. Biological denitrification, as a widely applied biochemical process, offers advantages of low cost and environmental compatibility. However, it is hampered by slow reaction kinetics, substantial carbon source demand, and challenges in controlling the treatment process, which can lead to unstable performance and residual sludge [14]. These limitations of conventional methods highlight a critical need for innovative, efficient, and sustainable technologies that can not only remove NO_3_^−^ effectively but also convert it into benign or even valuable products.

**Figure 1:**
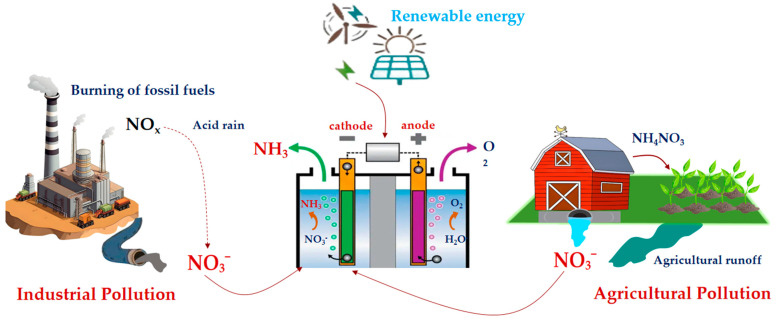
Application of eNO_3_RR for industrial and agricultural wastewater treatment.

Electrocatalytic nitrate reduction reaction (eNO_3_RR) has emerged as a highly promising alternative, leveraging electrons as clean reductants under ambient conditions [15,16]. This approach offers distinct advantages: (1) it eliminates the need for chemical additives, inherently preventing secondary pollution; (2) it allows for precise modulation of product selectivity (particularly towards valuable ammonia, NH_3_) through rational catalyst design and operational parameter optimization; (3) its modular reactor design features a small footprint and can be directly powered by renewable electricity (e.g., solar, wind), paving the way for carbon-neutral or even carbon-negative water treatment processes [17]. As illustrated in Figure 1, the eNO_3_RR enables the selective conversion of NO_3_^−^ from industrial and agricultural wastewater into NH_3_, achieving the dual objectives of environmental remediation and resource recovery.

The conversion of NO_3_^−^ to NH_3_ is particularly attractive from a circular economy perspective. NH_3_ is an indispensable chemical, serving as a cornerstone of modern agriculture through fertilizer production and holding great promise as a carbon-free energy carrier due to its high hydrogen content (17.6 wt%) and ease of liquefaction [18,19]. Currently, industrial-scale NH_3_ synthesis predominantly relies on the century-old Haber-Bosch (H-B) process, which operates under extreme conditions (350–500 °C, 150–350 bar) and accounts for approximately 1–2% of global energy consumption and 1% of global CO_2_ emissions annually [20]. The electrochemical nitrogen reduction reaction (NRR) is explored as a sustainable alternative for green NH_3_ synthesis from atmospheric N_2_ and water. However, its practical application is fundamentally challenged by the immense energy required to cleave the inert N≡N triple bond (941 kJ mol^−1^), the low solubility of N_2_ in aqueous electrolytes, and the overwhelming competition from the hydrogen evolution reaction (HER), collectively resulting in notoriously low NH_3_ yield rates and Faradaic efficiencies [21]. In stark contrast, eNO_3_RR bypasses the arduous N_2_ activation step. The higher solubility of NO_3_^−^ and its lower activation energy barrier often lead to significantly higher reaction rates and NH_3_ selectivity compared to NRR, making eNO_3_RR a more technologically and economically viable pathway for decentralized, sustainable NH_3_ production [22].

Despite these inherent advantages and considerable research progress in recent years [23,24,25], the practical implementation of eNO_3_RR faces significant scientific challenges. The reaction involves a complex multi-step mechanism encompassing eight-electron and nine-proton transfers, which leads to high activation barriers, sluggish kinetics, and a spectrum of possible reaction pathways that can yield various byproducts (e.g., NO_2_^−^, N_2_, N_2_O), thereby compromising the selectivity towards NH_3_ [26]. Moreover, the unavoidable competition from the HER further suppresses the Faradaic efficiency (FE) and yield of NH_3_ [27]. Therefore, a profound mechanistic understanding and the rational design of highly active, selective, and stable catalysts are paramount to overcoming these hurdles.

This review aims to provide a comprehensive and critical overview of the recent advances in electrocatalytic nitrate-to-ammonia conversion. We begin by elucidating the fundamental mechanisms and key reaction pathways of eNO_3_RR, followed by an in-depth discussion on the critical parameters governing performance, including the electrolyte microenvironment, applied potential, and reactor configuration. Further discussion of recent advances in catalysts, including single-metal catalysts, alloy catalysts, transition metal compounds, single-atom catalysts, carbon-based non-metal catalysts, and composite catalysts, highlights their significant roles in enhancing both the efficiency and selectivity. A distinctive feature of this review is its consistent assessment of catalyst strategies through the dual lenses of practicality and sustainable development. Finally, we outline the prevailing challenges and offer perspectives on future research priorities, aiming to guide the development of efficient, scalable, and commercially viable electrocatalytic systems for sustainable nitrogen management and green NH_3_ synthesis.

## 2. Mechanisms of eNO_3_RR to NH_3_

### 2.1. Fundamental Principles and Thermodynamic Considerations

**Figure 2:**
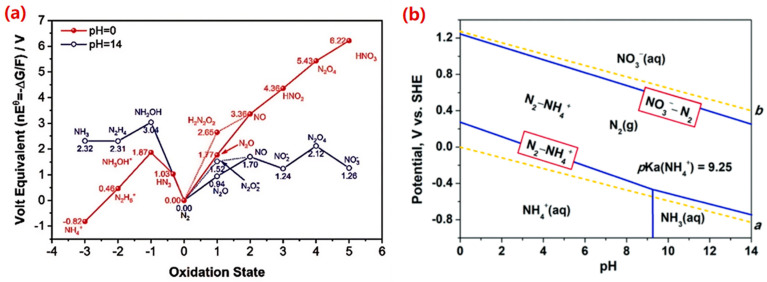
(**a**) Frost–Ebsworth diagram of nitrogen species in acid (pH = 0.0) and alkaline conditions (pH = 14.0) [29]; reproduced with permission Copyright 2020, Wiley-VCH GmbH. (**b**) Pourbaix diagram showing the reaction potential of nitrogen species and water in different values of the pH [30]. Reproduced with permission Copyright 2019, Royal Society of Chemistry.

The eNO_3_RR in aqueous solutions is a complex process involving multiple proton-coupled electron transfers (PCET) process, which can generate various products, including N_2_, N_2_O, NO, NH_2_OH, and NH_3_ [28]. Among these, N_2_ and NH_3_ represent the most thermodynamically stable products, as illustrated by the Frost-Ebsworth diagram (Figure 2a). This diagram reveals that a higher oxidizing potential corresponds to a steeper, more positive slope, indicating a greater thermodynamic driving force for the reduction process [29]. The corresponding reactions for the formation of N_2_ and NH_3_ are given in Equations (1) and (2), respectively:

NO_3_^−^ + 12H^+^ + 10e^−^ → N_2_ + 6H_2_O *E°* = 1.17 V vs. SHE (1)

NO_3_^−^ + 9H^+^ + 8e^−^ → NH_3_ + 3H_2_O *E°* = 0.69 V vs. SHE (2)

The thermodynamic landscape of eNO_3_RR is profoundly influenced by operational conditions, particularly pH. The speciation of the ammonia product (NH_3_ vs. NH_4_^+^) and the prevailing reaction pathways are highly pH-dependent. The Pourbaix diagram (Figure 2b) demonstrated that under standard conditions, the potential required for NH_3_ production via eNO_3_RR is very close to that of the competing HER (Equation (3)), leading to an inevitable kinetic competition that crucially impacts the FE for ammonia [30].

2H^+^ + 2e^−^ → H_2_(g), *E°* = 0 V vs. SHE (3)

Consequently, suppressing the HER kinetics is paramount for achieving high NH_3_ selectivity.

Despite the thermodynamic stability of N_2_ and NH_3_, by-products like N_2_O and NO are frequently observed experimentally. This discrepancy underscores that kinetic factors, rather than thermodynamics, often govern the product distribution. Furthermore, Pourbaix diagrams, based on bulk thermodynamics, do not account for critical catalyst surface effects such as local pH changes, electric double-layer structure, or adsorbate-adsorbate interactions. These factors can significantly alter proton transfer kinetics and cause the reaction pathway to deviate substantially from predictions under non-standard conditions [31]. Ultimately, the interplay between the thermodynamics and kinetics of NO_3_^−^ reduction and the competing HER dictates the overall current efficiency and selectivity of the process [32]. The HER not only consumes electrons and protons but also complicates the reaction network, thereby diminishing the selectivity and efficiency of eNO_3_RR.

### 2.2. Reaction Pathways of eNO_3_RR to NH_3_

**Figure 3:**
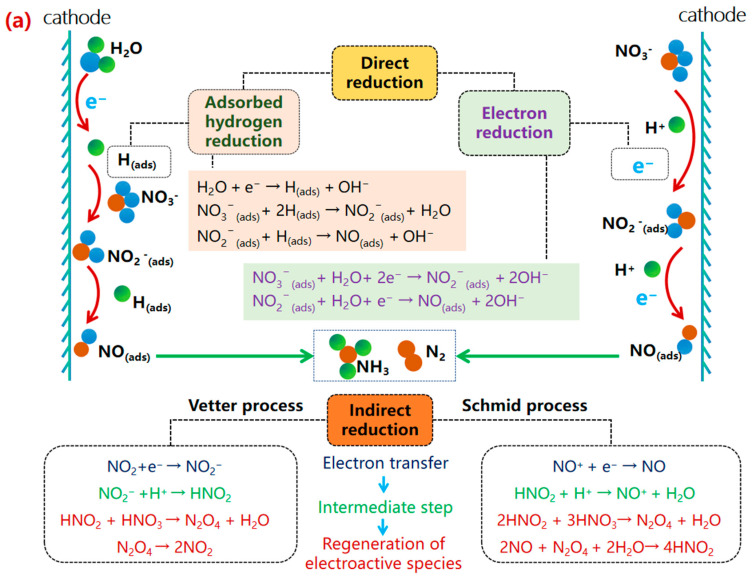

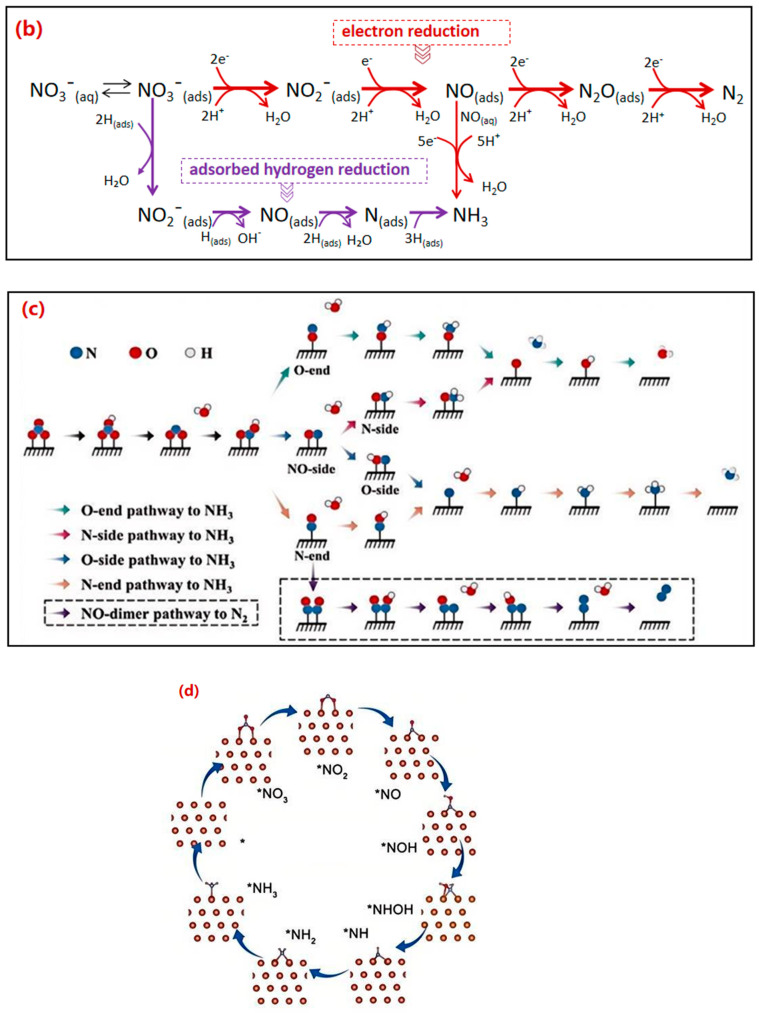
(**a**) Direct and indirect reaction pathways of eNO_3_RR [33]. Reproduced with permission Copyright 2023 Wiley-VCH GmbH. (**b**) The direct mechanism for electrochemical nitrate reduction mediated by electron reduction (red arrow) and adsorbed hydrogen reduction (purple arrow) [36]. Reproduced with permission Copyright 2021, ROYAL SOCIETY OF CHEMISTRY. (**c**) The detailed pathways of eNO_3_RR, including O-end, O-side, N-end, and N-side pathway to NH_3_, as well as NO-dimer pathway to N_2_O and N_2_ [45]. Reproduced with permission Copyright 2021, Wiley-VCH GmbH. (**d**) Schematic presentation of three pathways for nitrate reduction of Cu(111), here * denotes the adsorbed state of the substance [47]. Reproduced with permission Copyright 2021, American Chemical Society.

The eNO_3_RR process proceeds through a series of PCET steps, generating multiple nitrogen-containing intermediates. Two distinct mechanistic pathways have been extensively discussed: the indirect reduction and the direct reduction (Figure 3a) [33]. The fundamental distinction lies in the initial activation step: the direct mechanism involves electron transfer directly to the adsorbed NO_3_^−^ ion, while the indirect mechanism proceeds via homogeneous chemical reactions initiated by reactive nitrogen species (e.g., NO_2_) in solution, often exhibiting autocatalytic behavior [34].

#### 2.2.1. The Indirect Reduction Mechanism

The indirect reduction mechanism predominantly occurs under highly acidic conditions (>1.0 M) and high reactant concentration (1.0–4.0 M), where NO_3_^−^ exists predominantly as HNO_3_. Under these conditions, the reduction proceeds through critical intermediates such as NO^+^ and NO_2_, which initiate autocatalytic reactions, rather than through direct electron transfer to NO_3_^−^. The primary products of these cycles are NO_2_ and HNO_2_. These autocatalytic cycles can be categorized into the Vetter and Schmid pathways (Figure 3a) [33,35].

In the Vetter process, NO_2_ acts as the primary electroactive species. Its electroreduction generates NO_2_^−^, which rapidly protonates to form HNO_2_ under strong acidity. HNO_2_ then undergoes a fast chemical reaction with ambient HNO_3_ to form N_2_O_4_, which subsequently decomposes to regenerate two NO_2_ molecules. In the Schmid process, NO^+^ is reduced to NO, which then reacts directly with HNO_3_ to yield HNO_2_. The N_2_O_4_ supplied from the Vetter process can also react with NO to form HNO_2_. The resulting HNO_2_ may either decompose to regenerate NO^+^ (re-initiating the Schmid cycle) or participate in the Vetter cycle [35].

Although this pathway has been widely reported in early studies, it exhibits poor controllability and tends to generate numerous by-products, which is unfavorable for highly selective NH_3_ synthesis. For instance, NO_2_ and HNO_2_ produced via the Vetter and Schmid pathways can be readily further reduced to N_2_O or N_2_ rather than the target product NH_3_. Therefore, it is generally accepted in current research that the indirect mechanism is not conducive to eNO_3_RR aimed at NH_3_ synthesis, particularly under low NO_3_^−^ rate concentration conditions. Future studies should focus on developing catalysts that maintain high selectivity under mildly acidic or even neutral conditions to circumvent the drawbacks associated with the indirect pathway.

#### 2.2.2. The Direct Reduction Mechanism

The direct reduction pathway is more relevant for selective NH_3_ synthesis and can be further classified based on the reduction mediator: electron reduction and adsorbed hydrogen reduction (Figure 3b) [36]. This pathway generally involves three critical stages: NO_3_^−^ adsorption; reduction of NO_3_^−^ to NO_2_^−^ (often the rate-determining step); and reduction of NO_2_^−^ to NH_3_ or N_2_ (the selectivity-determining step).

(1) NO_3_^−^ adsorption

The eNO_3_RR process initiates with the adsorption of NO_3_^−^ onto the cathode surface (Equation (4)), a step crucial for activating the inert N–O bonds.

NO_3_^−^ ⇌ NO_3_^−^_(ads)_ (4)

The adsorption strength and configuration of NO_3_^−^ are influenced by the catalyst’s electronic structure and surface morphology. Factors such as electrode surface area, porosity, and the presence of competing ions in the electrolyte can significantly modulate the adsorption equilibrium and, consequently, the overall reaction rate [37,38].

(2) Reduction of NO_3_^−^ to NO_2_^−^: rate-limiting step

The NO_3_^−^_(ads)_ is initially reduced to NO_2_^−^_(ads)_ (Equation (5)) by the electrons from the electrode, which is normally considered as the rate-determining step (RDS) to regulate the reaction kinetics of the whole eNO_3_RR. The electron transfer into the lowest unoccupied molecular orbital (LUMO) of NO_3_^−^_(ads)_ needs to overcome a high-energy barrier, which can be accelerated by suitable electrodes [39,40]. It is noted that the 3d band energy of Cu is well compatible with the energy of the LUMO (π*) of NO_3_^−^. This electronic synergy endows Cu with superior capability to inject electrons directly into the N-O bonds of NO_3_^−^, promoting the reduction of NO_3_^−^ to NO_2_^−^ [41].

NO_3_^−^_(ads)_ + 2H^+^ + 2e^−^ → NO_2_^−^_(ads)_ + H_2_O (5)

(3) Electrochemical reduction of NO_2_^−^ to NH_3_: selectivity determining step

This process is described by the generic reaction (Equation (6)). Initially, NO_2_^−^_(ads)_ would be transformed into NO_(ads)_ through electron transfer (Equation (4)).

NO_2_^−^ + 5H_2_O + 6e^−^ → NH_3_ + 7OH^−^ (6)

NO_2_^−^_(ads)_ + 2H^+^ + e^−^ → NO_(ads)_ + H_2_O (7)

The subsequent fate of NO_(ads)_ represents the critical branch point leading either to the desired NH_3_ or to undesirable by-products like N_2_ and N_2_O [42,43,44]. The adsorption geometry of NO_(ads)_ (e.g., N-end, O-end, or side-on configuration) profoundly influences the reaction trajectory (Figure 3c) [45]. Multiple pathways have been proposed for the conversion of NO_(ads)_ to NH_3_. One common route involves sequential hydrogenation steps: NO_3_ → NOH_(ads)_ → NHOH_(ads)_ → NH_2_OH_(ads)_ → NH_3_ [46]. Based on systematic thermodynamic and kinetic analyses, Hu et al. proposed that the pathway NO_(ads)_ → NOH_(ads)_ → NHOH_(ads)_ → NH_(ads)_ → NH_2(ads)_ → NH_3(ads)_ is the most probable across a wide pH range (Figure 3d) [47]. Notably, these pathways share key intermediates (e.g., NOH_(ads)_, NHOH_(ads)_), underscoring their mechanistic relevance.

The hydrogenation steps can proceed via two primary modes:

One pathway is through stepwise protonation and hydrogenation (Equations (8)–(13)) [32]. The process begins with the adsorption of H_2_O on the electrode. When a potential is applied, adsorbed hydrogen atoms (H_(ads)_) are first generated by the Volmer step (Equation (10)) of the HER. This pathway is particularly relevant on precious metal-based catalysts such as Pd-based compounds that have a strong affinity for H [48].

H_2_O + e^−^→ H_(ads)_ + OH^−^ (Volmer) (8)

NO_(ads)_ + H_(ads)_ → 2N_(ads)_ + H_2_O (9)

N_(ads)_ + H_(ads)_ → NH_(ads)_ (10)

NH_(ads)_ + H_(ads)_ → NH_2(ads)_ (11)

NH_2(ads)_ + H_(ads)_ → NH_3(ads)_ (12)

NH_3(ads)_ → NH_3_ (13)

Another common pathway is via a series of sequential direct charge transfer reactions (Equations (14)–(17)) [49].

NO_(ads)_ + e^−^ + H^+^ → NOH_(ads)_ (14)

HNO_(ads)_ + e^−^ + H^+^ → H_2_NO_(ads)_ (15)

H_2_NO_(ads)_ + e^−^ + H^+^ → H_2_NOH (16)

H_2_NOH + 2e^−^ + 2H^+^ → NH_3_ + H_2_O (17)

Moreover, NO_(ads)_ can be desorbed from the surface to produce NO_(aq)_ (Equation (18)), followed by the generation of N_2_O_(ads)_ (Equation (19)). N_2_O_(ads)_ is further reduced to N_2_ (Equation (20)), or desorbed to produce N_2_O [49]. Particularly at higher potentials, the formation of N_2_O as a by-product is more favorable. By lowering the potential below 0.25 V vs. RHE, the thermodynamic driving force for the coupling of NO_(ads)_ and NO_(aq)_ species can be limited. Nonetheless, more negative potentials promote the competing hydrogen evolution reaction [50].

NO_(ads)_ → NO_(aq)_ (18)

NO_(ads)_ + NO_(aq)_ + 2H^+^ + 2e^−^ → N_2_O_(ads)_ + H_2_O (19)

N_2_O_(ads)_ + 2H^+^ + 2e^−^ → N_2_ + H_2_O (20)

It should be noted that two N_(ads)_ species could combine to form N_2_. However, energy barrier for N_(ads)_ diffusion (0.75 eV) is higher than that for H_(ads)_ transfer (0.10 eV). Due to the more favorable kinetics of H_(ads)_ migration, the formation of N-H bond is often preferred over N-N bond formation, which promotes NH_3_ production. Moreover, since H_(ads)_ participates in multiple steps, optimizing its generation and utilization is key to achieving efficient and selective NH_3_ synthesis. Researchers have developed innovative strategies, including introducing defects and vacancies to modulate the electronic structure of catalysts, which facilitates water activation and hydrogen species production.

In summary, the process of eNO_3_RR encompasses a key intermediate and two determining steps. The reduction of NO_3_^−^ to NO_2_^−^ is often the rate−determining step, while the subsequent conversion of NO_2_^−^/NO_(ads)_ to NH_3_ is the selectivity-determining step. The key intermediate is NO_(ads)_, which sits at a branching point leading either to desired NH_3_ or undesired N_2_/N_2_O byproducts. An ideal catalytic active site should facilitate the easy NO_3_^−^ adsorption and subsequent protonation while inhibiting N-N bond formation between NO_(ads)_ intermediates. The complexity of these multi-step PCET processes in eNO_3_RR necessitates the application of advanced in situ and operando techniques to identify reactive intermediates and true active sites, thereby enabling a deeper understanding of the reaction mechanisms and the validation of the pathways discussed above.

### 2.3. In Situ/Operando Characterizations

The accurate identification of reaction intermediates and the true active species of a catalyst is crucial for understanding electrocatalytic mechanisms [36]. To achieve this, in situ/operando techniques are indispensable, as they allow direct probing of key reaction intermediates, identification of active sites, and tracking of their dynamic evolution under realistic reaction conditions [51]. These methods enable real-time monitoring of the generation, transformation, and consumption of intermediates, as well as capturing dynamic changes in the structural and electronic properties of catalyst surfaces [52].

Elucidating the intricate reaction network of eNO_3_RR to ammonia requires a synergistic combination of advanced in situ characterization techniques. In situ Fourier Transform Infrared Spectroscopy (FTIR) provide molecular-level fingerprint information suitable for identifying surface-adsorbed species and intermediates1 [53,54,55]. For instance, Guo et al. [56] observed signals on a Pd/NF catalyst corresponding to intermediates such as −NH_2_ (~3336 cm^−1^), monodentate nitrite (~1470 cm^−1^), and NO^−^ (~1329 cm^−1^), supporting a sequential deoxygenation pathway from NO_3_^−^ to N_(ads)_. Similarly, Liu et al. [57] attributed signals at 1589, 1307, 1225, and 1145 cm^−1^ to NH_2_OH_(ads)_, NO_(ads)_, NO_2_^−^, and NH_2(ads)_, respectively, on Ni_1_Cu SAAO NWs; the stronger NH_2_OH_(ads)_ signal indicated enhanced deep hydrogenation capability. However, FTIR is limited by its penetration depth and potential interference from interfacial water, which can obscure certain intermediate signals.

In contrast to FTIR, in situ Raman spectroscopy is highly sensitive to phase and valence state changes, making it ideal for tracking the dynamic structural evolution of catalysts under operando conditions [58,59,60]. For example, Zhang et al. [61] reported the complete disappearance of Cu-O oxide Raman signals on CuCo NWs at reaction potentials, indicating in situ reduction to metallic Cu and Co as the active phases. Li et al. [62] further demonstrated the disappearance of Fe^3+^-O vibrations (407 and 609 cm^−1^) and the emergence of Fe^2+^-O peaks (201 and 275 cm^−1^), directly evidencing the critical Fe^3+^/Fe^2+^ redox cycle. Despite their strengths, vibrational spectroscopies like FTIR and Raman are generally not quantitative for volatile products [62,63].

This limitation is addressed by in situ differential electrochemical mass spectrometry (DEMS), which quantifies volatile intermediates and products by monitoring specific mass-to-charge ratios (*m*/*z*) [64]. Wang et al. [65] detected NO_2_ (*m*/*z* = 46), NO (*m*/*z* = 30), NH_2_OH (*m*/*z* = 33), and NH_3_ (*m*/*z* = 17) at the Cu/Cu_2_O interface, thereby reconstructing a pathway from N-O cleavage to stepwise hydrogenation. Luo et al. [66] used DEMS for quantitative comparison, revealing higher concentrations of HNO (*m*/*z* = 31) and NH_3_ (*m*/*z* = 17) over a Ru–Fe_2_O_3_ catalyst, thus confirming Ru’s role in promoting hydrogenation.

To further distinguish between direct electron transfer and hydrogen-mediated pathways, in situ electron paramagnetic resonance (EPR) spectroscopy provides unique evidence by detecting paramagnetic radicals. Roessler et al. [67] observed a significantly enhanced H_(ads)_ signal after introducing Ru into Fe_2_O_3_, supporting a hydrogen-mediated mechanism. Similarly, Liu et al. [68] demonstrated the consumption of H_(ads)_ radicals upon NO_3_^−^ addition via a radical quenching experiment, providing direct evidence for H_(ads)_ involvement in nitrate hydrogenation. In summary, the complementary use of these techniques constructs a complete experimental evidence chain, linking catalyst dynamics to reaction pathways and product formation.

The complexity of eNO_3_RR underscores the necessity of combining multiple in situ techniques for a holistic mechanistic understanding. For example, coupling FTIR with DEMS can correlate surface-bound intermediates with gaseous products. Future efforts should focus on improving temporal resolution to capture short-lived intermediates, enhancing spatial resolution for single-atom-level insights, and developing multimodal setups that integrate complementary techniques within a single operando cell. Moreover, the integration of machine learning for data analysis will be vital for extracting meaningful patterns from complex, multidimensional datasets. Looking ahead, emerging techniques such as ultrafast spectroscopy and synchrotron-based operando X-ray absorption spectroscopy (XAS) could provide unprecedented insights into transient species and electronic structure dynamics. These advancements will collectively accelerate the rational design of efficient catalysts for sustainable ammonia synthesis.

## 3. Factors Influencing the eNO_3_RR to NH_3_

The eNO_3_RR to NH_3_ is a complex process governed by multiple interrelated parameters. A fundamental understanding and precise optimization of these factors are paramount for enhancing the reaction efficiency, NH_3_ selectivity, and scalability for practical applications. This section provides a critical analysis of the key determinants of eNO_3_RR performance, focusing on the electrolyte microenvironment, the impact of applied potential (including innovative pulsed electrolysis), and the effect of reactor design.

### 3.1. Electrolyte Microenvironment

Beyond the pursuit of highly active catalysts, engineering the electrolyte microenvironment has emerged as a pivotal strategy for boosting eNO_3_RR performance. Major parameters such as the initial NO_3_^−^ concentration, electrolyte pH, and coexisting ions profoundly influence the local reaction conditions at the electrode-electrolyte interface, thereby dictating the overall activity, selectivity, and FE for NH_3_ production [69].

#### 3.1.1. Initial NO_3_^−^ Concentration

The initial adsorption of NO_3_^−^ onto the catalyst surface, a critical initiation step in eNO_3_RR, is often mass-transfer-limited. At low concentrations (<50 mg/L), the diffusion of NO_3_^−^ to the electrode surface becomes the rate-determining step, leading to diminished reaction rates [70,71]. Conversely, excessively high NO_3_^−^ concentrations (>500 mg L^−1^) may saturate active sites, impede the adsorption and desorption dynamics of key intermediates (e.g., NO_2(ads)_, N_(ads)_), and promote parasitic HER, consequently reducing both NH_3_ selectivity and FE [72].

Notably, an optimal concentration window exists. When the NO_3_^−^ concentration is maintained below 200 mg L^−1^, both high NO_3_^−^ conversion (>90%) and NH_3_ selectivity (>90%) can be achieved [73]. This observation aligns with the findings of Meng et al. [74], who reported superior NO_3_^−^ removal efficiency at concentrations below 200 mg L^−1^ compared to higher levels under identical conditions. The concentration-dependent performance underscores a delicate balance between mass transport and surface coverage, necessitating catalyst designs that optimize adsorption energetics across a realistic range of NO_3_^−^ concentrations.

Therefore, a comprehensive evaluation of eNO_3_RR performance requires systematic investigation across a range of NO_3_^−^ concentrations and buffer environments to elucidate intrinsic catalytic behavior and guide process optimization.

#### 3.1.2. Electrolyte pH

The electrolyte pH is a decisive factor that controls the reaction pathway and product distribution of eNO_3_RR by influencing the proton availability and the surface charge of the catalyst. Acidic conditions (pH < 7) favor the initial reduction of NO_3_^−^ to NO_2_^−^ but often lead to limited further conversion to NH_3_ [75]. The high proton concentration in acid facilitates the formation and accumulation of undesirable gaseous intermediates such as NO and N_2_O, reducing NH_3_ selectivity. In contrast, alkaline media (pH > 7) are generally more favorable for efficient NH_3_ synthesis, as they suppress the competing HER and stabilize key nitrogenous intermediates [76].

Moreover, pH directly modulates the electronic structure and local coordination environment of catalytic active sites. For instance, Co-MoS_2_ exhibits pH-dependent activity with a confined optimal operating potential window (−0.3 to −0.4 V vs. RHE) within pH 7–14, accompanied by enhanced structural stability and NH_3_ selectivity [77]. Similarly, a Cu_2_O-Cu/Ti composite electrode achieved an outstanding FE of 92% and an NH_3_ yield of 0.28 mmol cm^−2^ h^−1^ under alkaline conditions, whereas its performance deteriorated markedly under acidic conditions [78]. The enhanced performance in alkali can be attributed to a more favorable adsorption configuration of NO_3_^−^ and H_(ads)_, lower energy barriers for N-O bond cleavage, and suppressed H_2_ evolution.

In summary, optimizing the electrolyte pH, typically towards alkaline conditions, is essential for achieving high NH_3_ yield, selectivity, and catalyst durability. Future studies should aim to decouple the synergistic effects of pH from other parameters, such as electrolyte composition and catalyst structure, through advanced in situ/operando characterization.

#### 3.1.3. Coexisting Ions

Realistic nitrate-containing wastewater streams contain various anions (e.g., SO_4_^2−^, Cl^−^, CO_3_^2−^, PO_4_^3−^) and cations, which can significantly influence eNO_3_RR performance through competitive adsorption, complexation, or modulation of the interfacial electric field. Competitive adsorption between NO_3_^−^ and other anions can retard reaction kinetics. For example, Zhou et al. [79] demonstrated that increasing concentrations of carbonate, phosphate, and sulfate anions inhibit NO_3_^−^ adsorption and reduce its removal efficiency.

Cations play an equally critical, often overlooked, role in modulating reaction pathways and selectivity. Alkali metal cations (M^+^) can stabilize key oxygen-containing intermediates (e.g., NO_3_^−^_(ads)_, NO_2_^−^_(ads)_) via non-covalent electric-field effects, thereby altering the reaction energy landscape and enhancing NH_3_ selectivity. Using a copper catalyst, Wen et al. [80] showed that the performance trend follows the order Li^+^ < Cs^+^ < Na^+^ < K^+^. Through a combination of in situ techniques (EPR, FTIR, DEMS), they confirmed that K^+^ promotes more efficient proton-electron coupling with nitrogen-containing intermediates, facilitating the complete conversion of NO_3_^−^ to NH_3_. This cation effect is attributed to the weaker hydration energy of K^+^, allowing for a closer approach to the negatively charged cathode surface and a stronger interfacial electric field that stabilizes polar transition states.

These insights underscore the importance of tailoring electrolyte composition to optimize NH_3_ production. However, the complex interplay of ions in real wastewater matrices necessitates further systematic investigation using well-defined model systems and computational screening methods.

### 3.2. Impact of Applied Potential

**Figure 4:**
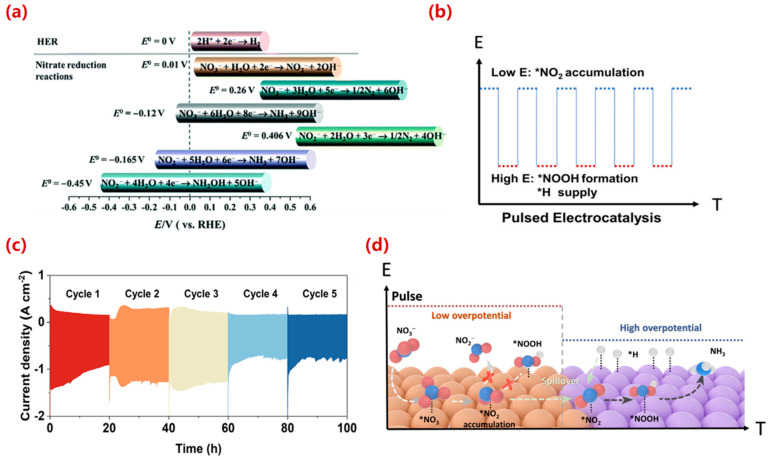
(**a**) The main reactions of eNO_3_RR and the thermodynamic potential of HER in aqueous solution at standard conditions [82]. Reproduced with permission Copyright 2020, American Chemical Society. (**b**) pulsed test schematic, (**c**) chrono-amperometry results of J-Cu@Co/NC with actual industrial current density under pulsed potentials, and (**d**) schematic illustration of ammonia electrosynthesis driven by pulsed potential [89]. Reproduced with permission Copyright 2025, Wiley-VCH GmbH. Here * denotes the adsorbed state of the substance.

The applied potential is a decisive operational parameter that controls the thermodynamics and kinetics of electron transfer, thereby dictating the product distribution of eNO_3_RR. As illustrated in Figure 4a [81], distinct major products are formed at different potentials, highlighting the profound impact of electrode potential on steering the reaction pathway [82].

#### 3.2.1. Potential-Dependent Product Selectivity

Shih et al. [83] systematically investigated the effect of overpotential, reporting a high N_2_ selectivity of 55.6% at a low overpotential (−0.6 V vs. Hg/HgO), while complete reduction of NO_3_^−^ to NH_4_^+^ was achieved at a higher overpotential (−1.2 V vs. Hg/HgO). A similar potential-dependent behavior was observed by Reyter et al. [84] over a Cu catalyst: NO_3_^−^ was primarily reduced to NO_2_^−^ at −0.9 V; within the range of −0.1 to −0.9 V, NO_2_^−^ was further reduced to hydroxylamine and eventually to NH_3_ during prolonged electrolysis; and direct conversion of NO_2_^−^ to NH_3_ became dominant at −1.1 V. This progression reflects the increasing driving force for multi-electron transfer at more negative potentials, enabling the deep hydrogenation required for NH_3_ synthesis.

Beyond altering product distribution, the applied potential also markedly enhances the reaction rate. For instance, Fang et al. [85] developed CuCo nanosheets that exhibited exceptional catalytic performance, demonstrating a strong correlation between increasing electrode potential (more negative) and improved NH_3_ yield. Wang et al. [86] further confirmed that NO_3_^−^ removal efficiency over Cu nanobelts increased with applied potential: after 30 min, NO_3_^−^ removal rates reached 24.2%, 56.3%, 91.5%, and 100% at applied potentials of −1.0, −1.2, −1.4, and −2.0 V, respectively.

Current density, a direct consequence of the applied potential, is another crucial parameter. Increasing the potential elevates the current density, enhancing the adsorption of NO_3_^−^ and had on the electrode surface and accelerating reaction kinetics. Hong et al. [87] demonstrated that higher applied voltages led to increased current densities and consequently higher NO_3_^−^ reduction rates. Similarly, Zhang et al. [88] reported nearly complete NO_3_^−^ removal within 60 min at current densities of 25 and 35 mA cm^−2^, while at 15 mA cm^−2^, the removal rate reached only 57.9% in 60 min. It is critical to note that while high current densities improve NO_3_^−^ conversion rates, they often reduce FE due to exacerbated HER and may aggravate electrode dissolution and corrosion. Therefore, selecting an appropriate cathode current density must balance the trade-off between NO_3_^−^ conversion rate, product selectivity, and overall energy efficiency.

#### 3.2.2. Pulsed Electrolysis: An Advanced Operational Strategy

Compared to constant-potential operation, dynamic electrochemical techniques such as pulsed electrolysis have emerged as a powerful strategy for enhancing eNO_3_RR performance by temporally decoupling reaction steps and mitigating mass transfer limitations.

A prominent example is the work by Li et al. [89], who designed a Cu@Co/NC catalyst combined with a dual-potential pulse protocol to achieve highly efficient conversion of NO_3_^−^ to NH_3_ (Figure 4b). Under pulsed conditions, the catalyst exhibited a remarkable FE of 98.32% and a production rate of 12.75 mg h^−1^ mgcat^−1^, significantly superior to performance under constant-potential conditions (FE ≈ 80%) (Figure 4c). The system also demonstrated exceptional stability, maintaining an FE above 90% after 100 h of operation under industrial current densities. DFT calculations revealed that the pulsed strategy optimizes the division of labor between Cu and Co sites: Cu stabilizes NO_2(ads)_ at lower potentials, while Co facilitates NOOH_(ads)_ formation at higher potentials, with interfacial charge transfer lowering the potential-determining step energy barrier to 0.62 eV (Figure 4d). This work provides a foundational mechanistic insight into the design of tandem catalytic systems via pulsed electrolysis, where different sites are selectively activated for specific steps.

Similarly, to address sluggish mass transfer and competing side reactions, Huang et al. [90] introduced a pulsed potential approach that achieved outstanding performance under mild conditions, including a high FE of 97.6%, a high NH_3_ yield of 2.7 mmol·h^−1^·mgRu^−1^, and a NO_3_^−^ conversion of 96.4%. In situ characterization and finite element analysis indicated that the periodic anodic potential pulse optimizes the adsorption configuration of key NO_(ads)_ intermediates and enhances the local NO_3_^−^ concentration by refreshing the diffusion layer.

In summary, the operating potential and the mode of electrolysis are critical factors governing the eNO_3_RR process. While higher potentials generally accelerate reduction rates, excessively high overpotentials can promote side reactions and reduce efficiency. Pulsed electrolysis offers a sophisticated means to overcome mass transfer limitations, enhance local reactant concentration, suppress the HER, and enable precise control over product formation. Future research should focus on optimizing pulse waveforms (frequency, duty cycle) and integrating real-time feedback control for adaptive operation, paving the way for intelligent electrocatalytic synthesis.

### 3.3. Effect of Reactor Structure

The architecture of the electrochemical reactor critically influences the efficiency, selectivity, and scalability of the eNO_3_RR by governing mass transport, product separation, and current distribution.

The most common configurations include single-chamber cells (SCCs) and dual-chamber cells (DCCs) separated by an ion-exchange membrane. In an SCC, the proximity of the anode and cathode allows reduction products like NH_3_ to diffuse to the anode and be re-oxidized, significantly decreasing the FE. In contrast, a DCC, typically employing a cation-exchange membrane (CEM), mitigates these undesired cross-over reactions by physically separating the compartments, thereby enhancing product selectivity and FE [91]. Ding et al. [92] compared the performance of SCC and DCC configurations using a graphite felt cathode, finding a NO_3_^−^ removal rate of only ~10% in the SCC compared to >75% in the DCC. The H-type cell, separated by a Nafion membrane, is a widely adopted DCC design in fundamental studies. However, the reliance on an ion-exchange membrane introduces additional ohmic resistance and cost, while also being susceptible to fouling in complex wastewater streams.

To advance towards practical application, researchers have developed novel reactor designs emphasizing enhanced mass transfer and in situ product recovery. Flow cell systems have attracted significant attention due to their low energy consumption, high mass transfer efficiency (enabled by forced convection), and suitability for long-term continuous operation. These systems often employ high-surface-area electrodes (e.g., gas diffusion electrodes, GDEs) and optimized flow fields.

For simultaneous NH_3_ production and capture, Mi et al. [93] designed an integrated “two-in-one” flow cell electrolyzer combining the eNO_3_RR reaction chamber with a GDE for NH_3_ capture. This design demonstrated superior performance, achieving an FE of 90.2% and an NH_3_ production rate of 2.1 mmol·h^−1^·cm^−2^, outperforming conventional H-type cells. The flow cell configuration with a GDE not only accelerates NO_3_^−^ reduction by ensuring sufficient reactant supply but also suppresses the HER by modulating the local proton concentration and creating an optimized triple-phase boundary.

For resource recovery-oriented treatment, Zhou et al. [94] developed a renewable energy-driven, filtration-coupled device that enhances NO_3_^−^ mass transfer and enables in situ recovery of NH_3_ through a hydrophobic gas-permeable membrane without the need for pH adjustment. Using a Cl-Cu monolithic electrode, the device achieved stable operation for 100 h in wastewater containing 50 mg·L^−1^ NO_3_^−^-N, with an NH_3_ recovery rate of 420 μg·h^−1^·cm^−2^. The integration of reaction and separation units represents a key step towards economically viable and energy-efficient nitrogen management.

Despite considerable progress, challenges remain in reactor design, including the cost and long-term stability of membranes, mass transfer limitations and gas bubble management under high current densities, and ensuring fluid distribution uniformity in large-scale cells. Future research should integrate multi-physics simulations (e.g., computational fluid dynamics) with intelligent control strategies to optimize reactor architecture and operational conditions, ultimately facilitating the industrialization of eNO_3_RR technology.

## 4. Catalysts for eNO_3_RR to NH_3_

### 4.1. Criteria for Selection of Catalysts

Several parameters are employed to evaluate the performance of catalysts for eNO_3_RR to NH_3_, such as NO_3_^−^ conversion efficiency, NH_3_ selectivity, FE, energy efficiency (EE), and NH_3_ yield rate. The following equations (Equations (21)–(23)) are used to calculate these parameters [95].

NO_3_^−^ conversion efficiency (%). This parameter represents the fraction of the initial NO_3_^−^ that has been consumed during the electrolysis. It is calculated using the initial and the final molar concentrations of NO_3_^−^.

(21) $$Conversion=\left[\frac{c_{0}\left(NO_{3}^{-})-c_{t}(NO_{3}^{-}\right)}{c_{0}(NO_{3}^{-})}\right]\times 100\%$$

Here, $c_{0}\left(NO_{3}^{-})\;\mathrm{and}\;c_{t}(NO_{3}^{-}\right)$ are the ${NO}_{3}^{-}$. concentrations (mol L^−1^) before and after electrolysis, respectively.

NH_3_ selectivity (%). This parameter represents the molar percentage of the consumed ${NO}_{3}^{-}$. that is selectively converted into NH_3_, reflecting the catalyst’s ability to steer the reaction away from undesired by-products.

(22) $$Selectivity=\left[\frac{C_{NH_{3}}}{c_{0}(NO_{3}^{-})-c_{t}(NO_{3}^{-})}\right]\times 100\%$$

Here, $C_{NH_{3}}$ is the concentration of produced NH_3_ (mol L^−1^), and $c_{0}(NO_{3}^{-})-c_{t}(NO_{3}^{-})$ is the change in ${NO}_{3}^{-}$. concentration (mol L^−1^).

FE (%): This is the paramount metric for electron economy, representing the percentage of the total passed charge utilized for the eight-electron reduction of NO_3_^−^ to NH_3_.

(23) $$FE\;=[(\frac{(8\times F\;\times \;C_{NH_{3}}\times V)}{M_{NH_{3}}\times Q}]\;\times \;100\%$$

Here, F is the Faraday constant (96,485 C mol^−1^), $M_{NH_{3}}$ is the molecular mass of NH_3_ (M = 17 g mol^−1^), and Q is the total charge passed through the electrode.

Half-Cell energy efficiency (EE, %). This metric is a crucial indicator for assessing the economic viability of the catalytic process, as it quantifies the energy cost of producing NH_3_. It is defined as the ratio of the theoretical minimum electrical energy required to produce NH_3_ (based on the reaction’s thermodynamic equilibrium potential) to the actual electrical energy consumed by the full electrochemical cell. A higher energy efficiency indicates a more economically feasible process.

(24) $$EE=\frac{{(E}_{\mathrm{OER}\;}^{{}^{\circ}}-\;E_{NH_{3}\;}^{{}^{\circ}})\times FE_{NH_{3}}}{{(E}_{\mathrm{OER}\;}-\;E_{NH_{3}})}\times 100\%$$

Here, $E_{\mathrm{OER}\;}^{{}^{\circ}}$ is the equilibrium potential of OER (1.23 V vs. RHE), $E_{{\mathrm{NH}}_{3}}^{{}^{\circ}}$ is the equilibrium potential of ${NO}_{3}^{-}$ electroreduction to NH_3_ (0.69 V vs. RHE), FE (NH_3_) is the FE for NH_3_, and $E_{\mathrm{OER}}$ and $E_{NH_{3}}$ are the applied potentials.

Yield Rate. The yield rate quantifies the production speed of NH_3_.

(25) $$\mathrm{Yild}\;\mathrm{Rate}=\frac{C_{NH_{3}}\times V}{t\times A}$$

(26) $$\mathrm{Yild}\;\mathrm{Rate}=\frac{C_{NH_{3}}\times V}{t\times m}$$

Here, $C_{NH_{3}}$ is the concentration of NH_3_ (${\mathrm{mol}}^{-1}$), t is the electrolysis time (s or h), V is the volume of electrolytes (L), A is the geometric area of the electrode (cm^2^), m is the mass loading of the catalyst (mg).

### 4.2. Advances in Different Types of Catalyst Research

**Figure 5:**
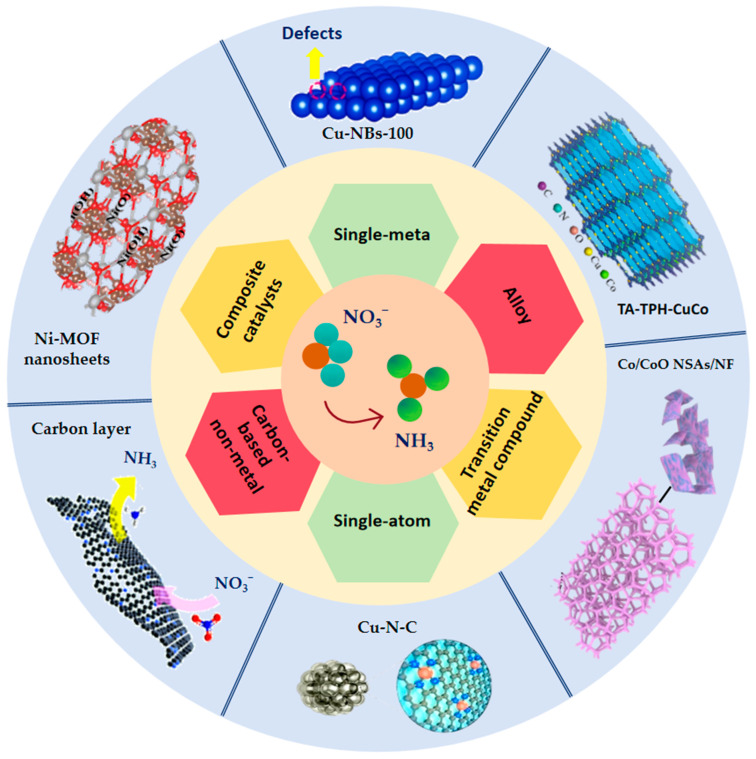
Different types of catalysts for eNO_3_RR to NH_3_ in our study.

Catalysts play a crucial role in the selective conversion of NO_3_^−^ to NH_3_, directly influencing the efficiency of NH_3_ production. Over the past decades, a wide variety of catalysts including metal- and non-metal-based materials, have been investigated for eNO_3_RR. Both experimental and theoretical calculations have suggested that noble metals (e.g., Au, Ag, Pd, Ru, and Rh) possess high corrosion resistance and exhibit outstanding activity for eNO_3_RR [96,97,98,99,100,101]. These metals typically feature higher d-orbital energy levels and coordination numbers, which provide abundant reactive sites. However, their high cost and scarcity remain significant challenges for widespread application. Therefore, the development of non-precious metal-based catalysts is essential for advancing eNO_3_RR to NH_3_ synthesis. In the following sections, we discuss recent progress in various catalytic systems, including single-metal catalysts, alloy catalysts, transition metal compounds, single-atom catalysts, carbon-based non-metal catalysts, and composite catalysts for eNO_3_RR (Figure 5).

#### 4.2.1. Single-Metal Catalysts

Single-metal catalysts serve as a fundamental platform for deciphering the structure-activity relationships in eNO_3_RR due to their relatively simple composition and well-defined active sites. Among numerous non-noble metal-based catalysts, copper (Cu)-based catalysts are widely employed for eNO_3_RR owing to their ability to effectively regulate the critical step of NO_3_^−^ reduction to NO_2_^−^. The catalytic advantage stems from an intrinsic electronic structure match with the reactant: the similarity between the d-orbital energy levels of Cu and the lowest unoccupied molecular orbital (LUMO) π* of NO_3_^−^ facilitates electron transfer from the metal to the antibonding orbital of NO_3_^−^, thereby weakening the N-O bonds and promoting the initial adsorption and activation of NO_3_^−^ [102,103,104]. Additionally, Cu-based catalysts possess relatively low intrinsic activity for the HER, effectively suppressing this competing side reaction.

**Figure 6:**
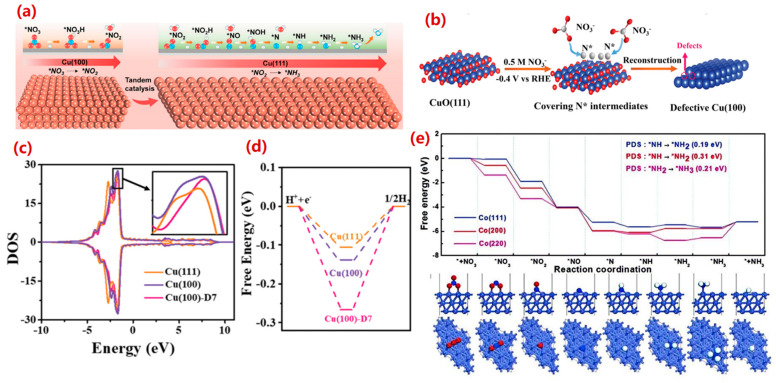
(**a**) Tandem interaction of Cu(100) and Cu(111) facets [105]. Reproduced with permission Copyright 2020, Elsevier Ltd. (**b**). Schematic illustration of eNO_3_RR intermediate adsorption manipulating the exposed facet of Cu during the reduction of CuO(111) nanobelts. and (**c**) Computed density of states (DOS) of Cu and (**d**) free adsorption energy of H_(ads)_ intermediates on the models of Cu(111), Cu(100), and Cu(100)-D7 [106]. Reproduced with permission Copyright 2021, Royal Society of Chemistry. (**e**) Free energy curves of NO_3_^−^ reduction reaction on Co(111), Co(200) and Co(220) crystal planes, and atomic configurations of the reaction process on the Co(111) surface (side view and top view) [108]. Reproduced with permission Copyright 2022, Royal Society of Chemistry. Here * denotes the adsorbed state of the substance.

Recently, various nanostructured Cu materials have demonstrated remarkable catalytic performance for eNO_3_RR. Fu et al. [105] synthesized Cu nanosheets exposing the (111) facet for the electrocatalytic reduction of NO_3_^−^, achieving an NH_3_ formation rate of 390.1 mg·mg^−1^·h^−1^ and a FE of 99.7%. This excellent performance was attributed to the suppression of HER and a significant enhancement in the rate of the rate-determining step on the Cu(111) facet. Density functional theory (DFT) calculations suggested a tandem mechanism, where the NO_2_^−^ intermediate was initially generated on the Cu(100) facets and subsequently hydrogenated on the Cu(111) facets (Figure 6a). In a complementary study, Hu et al. [106] synthesized rough Cu nanoribbons enriched with Cu(100) facets via in situ electrochemical reduction of ultrathin Cu oxide nanoribbons under eNO_3_RR conditions. This defective Cu(100) surface delivered a high NH_3_ yield rate of 650 mmol·h^−1^·gcat^−1^, which is 2.3-fold higher than that of the Haber-Bosch process. Combined experimental and DFT studies revealed that the Cu(100) facets and their surface defects caused an upward shift of approximately 0.26 eV in the d-band center, effectively reducing the reaction energy barrier for the NH_2_→NH_3_ step (ΔG = 0.18 eV) (Figure 6c). Simultaneously, the strong adsorption energy for H_(ads)_ intermediates significantly suppressed the competing HER (Figure 6d). These studies highlight the distinct roles of different Cu facets: Cu(100) and its defects are beneficial for key hydrogenation steps, while Cu(111) may suppress HER or participate in tandem reactions under specific conditions.

Beyond Cu, iron-group elements (e.g., Fe, Co, Ni) also demonstrate significant potential. Taking Co-based catalysts as an example, Deng et al. [107] reported that a Co-NAs electrode achieved a high current density of −2.2 A·cm^−2^ and an NH_3_ production rate of 10.4 mmol·h^−1^·cm^−2^ at −0.24 V vs. RHE. This performance was attributed to the high intrinsic activity of Co^0^, intimate contact with the conductive substrate, and a nanostructure exposing numerous active sites. The intrinsic mechanism involved a synergy between the deprotonation of H_2_O molecules and the hydrogenation of key intermediates on the catalyst surface, which significantly reduced the reaction kinetic barrier to 0.2 eV, markedly lower than that of the traditional Volmer step. Chen et al. [108] reported Co-NCNT nanohybrids supported on carbon paper as a highly active electrocatalyst, achieving a high NH_3_ yield of 5996 μg·h^−1^·cm^−2^ and an FE of 92% in alkaline electrolytes. DFT calculations on different Co crystal planes indicated that Co(111) was the most active surface for eNO_3_RR (Figure 6e).

In summary, single-metal catalysts, characterized by uniform active sites, greatly facilitate the mechanistic analysis of complex reaction pathways. Their well-established, controllable preparation methods also ensure high reproducibility, which is a cornerstone of rigorous fundamental studies. Nevertheless, the limited tunability of their electronic structure prevents the simultaneous optimization of adsorption strengths for NO_3_^−^ intermediates and H_(ads)_, causing substantial current loss to the competing HER. This drawback is exacerbated under realistic wastewater conditions, where wide variations in NO_3_^−^ concentration impede the consistent high performance of a single active site. Therefore, to overcome these inherent limitations, strategies such as alloying and designing single-atom catalysts have emerged as promising pathways to enhance the performance of metallic catalysts.

#### 4.2.2. Alloy Catalysts

Alloy catalysts emerge as a promising strategy to address these challenges by enabling precise modulation of the adsorption energies for key intermediates through intermetallic electronic effects (e.g., ligand and strain effects) and synergistic interactions. While early studies predominantly utilized precious-metal-based alloys (e.g., Pd-Cu, Cu-Pt) [109,110,111,112] to enhance activity and selectivity, their practical application is constrained by cost and scarcity. Recent advancements have thus shifted focus towards non-precious multimetallic alloys, where the strategic combination of elements can create synergistic effects, optimizing different steps within the eNO_3_RR pathway for superior overall performance [113].

**Figure 7:**
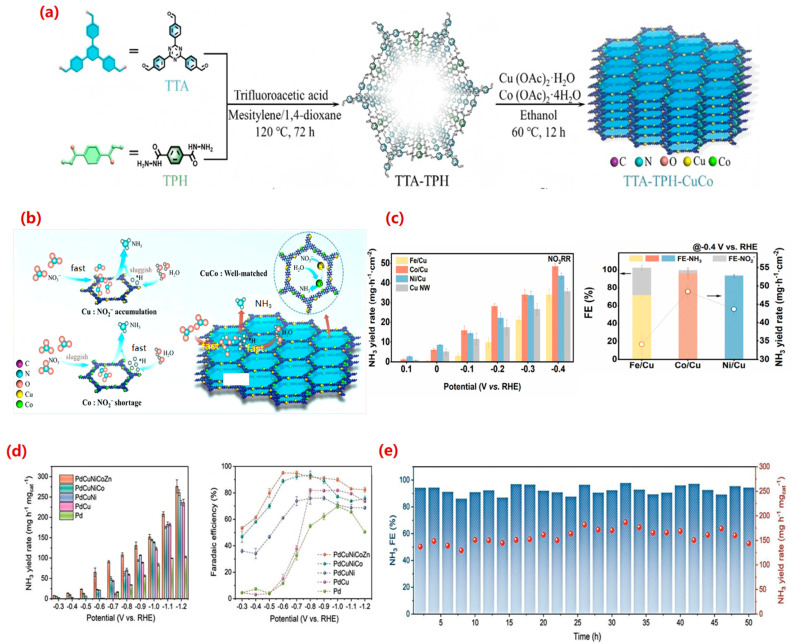
(**a**) The schematic diagram of preparing TTA-TPH and TTA-TPH-CuCo. (**b**) The schematic illustration of the tandem mechanism on TTA-TPH-CuCo [114]. Reproduced with permission 2025 Copyright Wiley-VCH. (**c**) NH_3_ yield rate and FE of catalysts [115]. Reproduced with permission 2025 Copyright Wiley-VCH. (**d**) NH_3_ yield rate and FE of high-entropy metallene catalyst and (**e**) consecutive recycling test over high-entropy metallene [117]. Reproduced with permission 2025 Copyright Nature. Here * denotes the adsorbed state of the substance.

A prominent design principle for alloy catalysts involves constructing tandem catalytic systems, where different metal sites preferentially catalyze sequential steps. This concept is effectively demonstrated in the work of Zhong et al. [114], who synthesized a hydrazone-linked covalent organic framework (COF) with atomically dispersed Cu and Co sites (TTA-TPH-CuCo) (Figure 7a). This catalyst achieved a remarkable NH_3_ yield of 20.8 mg·h^−1^·cm^−2^ and a high FE of 92.16% at −0.75 V vs. RHE, significantly outperforming its single-metal counterparts. The high performance was attributed to a tandem mechanism: Cu sites, with their strong affinity for NO_3_^−^, facilitate the initial reduction of NO_3_^−^ to NO_2_^−^, while adjacent Co sites enhance the adsorption and supply of active hydrogen, promoting the subsequent conversion of NO_2_^−^ to NH_3_. This spatial decoupling of reaction steps mitigates the accumulation of toxic nitrite intermediates and suppresses HER (Figure 7b). Further extending this paradigm, Yan et al. [115] systematically integrated iron-group nanosheets (Fe, Co, Ni) with Cu nanowires. Their study provided crucial insights into the role of specific metals, revealing that Cu and Fe sites are highly effective for the NO_3_^−^ to NO_2_^−^ step, whereas Co and Ni sites excel at the critical NO_2_^−^-to-NH_3_ conversion. Among these, the Cu-Co tandem system achieved an exceptional FE of 96.46% and a high NH_3_ yield of 48.44 mg·h^−1^·cm^−2^ at a relatively low potential of −0.4 V vs. RHE, underscoring the importance of matching metal properties with specific reaction steps (Figure 7c).

Beyond spatial tandem effects, precise electronic structure modulation at the atomic level offers another powerful avenue for enhancing eNO_3_RR. Cai et al. [116] exemplified this by embedding single Ni atoms into a Cu catalyst. This Ni-alloyed Cu catalyst achieved complete conversion of NO_3_^−^ to NH_3_ with a near-unity FE of 100% and a yield rate of 326.7 μmol·h^−1^·cm^−2^ at −0.55 V vs. RHE, which is approximately 10.7 times higher than that of pure Cu. Density functional theory (DFT) calculations revealed that the isolated Ni atoms significantly modulate the electronic structure of neighboring Cu atoms, optimizing the adsorption energy of the key intermediate NOOH_(ads)_. This electronic optimization lowers the energy barrier for the rate-determining protonation step, thereby accelerating the overall reaction kinetics while effectively suppressing HER and by-product formation. This work highlights the potential of single-atom alloy designs for achieving ultimate atomic efficiency and selectivity.

Recently, high-entropy alloys (HEAs), comprising five or more elements, have opened new frontiers in catalyst design by leveraging the so-called “cocktail effect.” Zhang et al. [118] first reported the application of a HEA (FeCoNiAlTi) for eNO_3_RR. Through a phase engineering strategy that introduced intermetallic nanoparticles, they increased the electrochemical active surface area and charge transfer efficiency, resulting in an FE of 95.23%. Subsequent experimental and theoretical analyses identified Fe, Co, and Ni as the primary active sites, suggesting a multi-site cooperative mechanism. In a complementary study, Zhou et al. [117] have prepared a high-entropy alloy catalyst consisting of Pd, Cu, Ni, Co, and Zn elements through the pyrolysis of a high-entropy metallene. Through multimetal interactions, various active centres are formed and sufficiently exposed over the metallene. Each element performs its own duties and jointly lowers the energy barrier of the rate-determining step. Optimizing the elemental proportions, the HEA catalyst exhibited exceptional eNO_3_RR activity with high FE of 99.0% and high NH_3_ yield rate of 447 mg h^−1^ mg^−1^ in a strongly alkaline electrolyte (Figure 7d). Its comprehensive catalytic performance significantly outperforms traditional single-atom and binary/ternary alloy catalysts. It is noted the HEA catalyst can maintain high activity and structural stability throughout the 50 h period (Figure 7e).

In summary, alloy catalysts, ranging from bimetallic systems to complex HEAs, demonstrate immense potential for advancing eNO_3_RR by enabling sophisticated control over reaction pathways and intermediate adsorption energies. Tandem catalysts decouple complex reactions, while atomic-level electronic tailoring optimizes key steps. HEAs further expand this paradigm by offering a platform for continuous electronic tuning. Despite promising progress, significant challenges remain. The stability of these complex structures under operational conditions, especially for HEAs with potential elemental segregation, requires thorough investigation. The scalable synthesis of structurally well-defined alloys, particularly atomically dispersed or HEA catalysts, must be addressed for practical application. Furthermore, a deeper mechanistic understanding gained through in situ/operando characterization and standardized activity metrics is essential to guide the rational design of next-generation alloy catalysts for sustainable NH_3_ synthesis.

#### 4.2.3. Transition Metal Compound Catalysts

Transition metal compounds (including oxides, sulfides, borides, and phosphides) have emerged as promising catalysts for the eNO_3_RR to NH_3_. Their catalytic activity originates from the hybridization between metal d-orbitals and non-metal p-orbitals, which effectively modulates the electronic configuration of active sites, thereby optimizing the adsorption and activation of NO_3_^−^ species.

(1) Transition metal oxide catalysts

Transition metal oxides represent a class of highly efficient catalysts for eNO_3_RR, primarily due to their tunable electronic structures and the prevalence of oxygen vacancies, which serve as active sites for NO_3_^−^ adsorption and activation. This section systematically reviews the eNO_3_RR performance of typical transition metal oxides, such as copper oxides (CuO, Cu_2_O), iron oxides (Fe_3_O_4_), and cobalt oxides (CoO).

Given the inherent NO_3_^−^ reduction capability of metallic Cu, Cu-based oxides have been extensively investigated [119,120]. For instance, Li et al. [121] synthesized spherical CuO nanoparticles enriched with oxygen vacancies. This catalyst achieved an impressive NH_3_ yield rate of 15.53 mg h^−1^mgcat^−1^ and a high FE of 90.69%. The DFT calculations revealed that the in situ formation of Cu(OH)_2_ enhanced NO_3_^−^ adsorption and suppressed the competing hydrogen evolution reaction (HER), while the exposed Cu (111) facets facilitated the hydrogenation steps. This synergy between Cu(OH)_2_ and Cu sites significantly promoted the conversion of NO_3_^−^ to NH_3_. In another study, Wang et al. [65] prepared CuO nanowire arrays via thermal treatment of Cu(OH)_2_ precursors. Under electrochemical conditions, these arrays underwent reconstruction into Cu-Cu_2_O heterostructures, which served as the active phase for nitrate-to-ammonia conversion, delivering an NH_3_ yield of 0.2449 mmol h^−1^cm^−2^ and a high FE of 97%, with an NH_3_ selectivity of 81.2%. DFT calculations indicated that the Cu-Cu_2_O interface promoted electron transfer, stabilizing the key NOH_(ads)_ intermediate and minimizing HER competition.

Driven by the demand for sustainable NH_3_ synthesis, earth-abundant iron-based oxides have gained recent attention [122]. Fan et al. [123] reported the in situ growth of Fe_3_O_4_ particles on stainless steel, which exhibited superior electrical conductivity and stability. The catalyst achieved a remarkable FE of 91.05% and an NH_3_ yield of 10,145 μmol h^−1^cm^−2^. RHE, maintaining stable performance for over 12 h. Theoretical studies identified the Fe_3_O_4_(311) facets as highly active for NO_3_^−^ binding, with the hydrogenation of NO_2(ads)_ to NO_2_H_(ads)_ being the potential-determining step (ΔG = 0.13 eV). Similarly, Yu et al. [124] constructed heterostructured Co/CoO nanosheet arrays (Co/CoO NSAs), which achieved an FE of 93.8% and an NH_3_ yield of 194.46 μmol h^−1^ cm^−2^. The enhanced performance was attributed to the electron-deficient Co sites induced by Schottky contact at the Co/CoO interface, which not only suppressed HER but also increased energy barriers for by-product formation.

(2) Other metal compound catalysts

Beyond oxides, other transition metal compounds (e.g., phosphides, borides, and sulfides) have shown great potential in eNO_3_RR, benefiting from the synergistic effects between metal and non-metal elements. Ye et al. [125] pioneered the use of cobalt phosphide nanosheet arrays on carbon cloth (CoP NAs/CFC), which achieved a near-unity FE (~100%) and a high NH_3_ yield of 9.56 mol h^−1^ m^−2^. The incorporation of phosphorus was crucial for stabilizing the active phase and reducing the energy barrier of the rate-determining step, as confirmed by DFT calculations. Zhang et al. [126] explored iron boride (FeB_2_, a representative MBene) as an eNO_3_RR catalyst, reporting an FE of 96.8% and an NH_3_ yield of 25.5 mg h^−1^ cm^−2^. Mechanistic studies revealed a unique division of labor: B sites adsorbed and activated NO_3_^−^, while Fe sites dissociated H_2_O to generate H_(ads)_. Subsequent hydrogen spillover from Fe to B sites accelerated the hydrogenation of intermediates. Wang et al. [127] synthesized MoS_2_ with sulfur vacancies (SVs) via a one-step hydrothermal method. The SVs were shown to lower the energy barrier of the potential-determining step, and the presence of H_(ads)_ on Mo sites coupled with N_(ads)_ species to enhance activity.

Above all, metal compound catalysts, mainly including oxides, sulfides, and phosphides, have also attracted widespread interest owing to their abundant reserves and tunable electronic structures. Among them, metal oxides demonstrate considerable catalytic potential due to their excellent NO adsorption capacity and significant ion exchange characteristics. Additionally, their mature preparation processes and ease of scalability offer notable economic advantages and industrialization prospects. However, most metal oxide semiconductors suffer from poor electrical conductivity, which hampers interfacial charge transfer efficiency. Moreover, under prolonged operation in strong electric fields and reactive environments, they are prone to chemical reduction or anodic dissolution, leading to activity degradation. In recent years, metal nitrides and phosphides have shown potential as alternatives to oxides, owing to their metal-like high electrical conductivity and corrosion resistance.

#### 4.2.4. Single-Atom Catalysts

Reducing the size of metal particles to the single-atom scale is a promising strategy for enhancing atom utilization efficiency and achieving unique activity and selectivity in eNO_3_RR to NH_3_ [128]. By enabling precise tuning of the coordination environment around the metal centers, single-atom catalysts (SACs) can optimize the adsorption behavior of reaction intermediates, thereby effectively promoting the conversion pathway of NO_3_^−^ to NH_3_ while suppressing side reactions [129,130,131,132,133,134,135]. This section focuses on recent advances in non-precious metal-based SACs (particularly d-block transition metals) for eNO_3_RR, highlighting the decisive role of coordination engineering and electronic structure modulation in determining their catalytic performance [136,137].

Among various SACs, Cu-based single-atom catalysts, especially nitrogen-coordinated Cu/NC materials, have demonstrated exceptional eNO_3_RR performance. For instance, Zhu et al. [138] synthesized a metal-nitrogen-carbon (M-N-C) electrocatalyst (Cu-N-C-800) consisting of carbon nanosheets embedding isolated copper atoms coordinated with nitrogen. Their results revealed that Cu species coordinated with N, particularly in a Cu-N_2_ configuration, are crucial for the favorable adsorption of NO_3_^−^ and NO_2_^−^. This strong adsorption enhanced the rate of NO_3_^−^ conversion to NH_3_ and N_2_, ultimately achieving a high FE of 96.8% for NH_3_ production. These findings underscore the potential of Cu SACs for NO_3_^−^ reduction. Complementarily, Yin et al. [129] demonstrated that the electronic structure of the Cu-N_4_ coordination environment inhibits the formation of N_2_, N_2_O, and H_2_, while facilitating orbital hybridization between the 2p orbitals of NO_3_^−^ and the 3d orbitals of the Cu single-atom sites, thereby directing the reaction towards highly selective NH_3_ synthesis.

Fe-based single-atom catalysts have also exhibited high selectivity and efficiency. Xu et al. [134] developed an iron single-atom catalyst coordinated with nitrogen and phosphorus on a hollow carbon polyhedron (Fe-N/P-C). The introduction of phosphorus atoms broke the local charge symmetry of the single-Fe sites, facilitating the adsorption of NO_3_^−^ and the enrichment of key reaction intermediates. Experimental results showed that the Fe-N/P-C catalyst achieved an FE of 90.3% and an NH_3_ yield of 17,980 μg h^−1^ mgcat^−1^. In another study, Li et al. [139] prepared N-coordinated Fe sites with atomic-level dispersion on a carbon support. The electroactive iron sites in the isolated atomic state exhibited a turnover frequency twelve times higher than that of metallic iron particles.

Recently, Co single-atom catalysts have emerged as promising materials, attracting growing research interest. Yang et al. [135] synthesized electron-deficient Co nanocrystals (Co/PN-C) via Co-pyridinic N interaction to promote electrocatalytic NO_3_^−^ reduction to NH_3_. DFT calculations indicated that the PN-C support possesses strong electron-withdrawing capability, creating electron-deficient sites on the metallic Co through interfacial electron depletion. These electron-deficient Co centers concurrently enhanced the adsorption/activation of NO_3_^−^ and optimized the adsorption of NH_(ads)_, thus promoting the hydrogenation step. This synergistic effect reduced the energy barrier of the rate-determining step, favoring selective NH_3_ generation.

In summary, SACs are notable for their high atom utilization, activity, and selectivity in nitrate reduction. Moreover, SACs can enhance intermediate adsorption and conversion while minimizing competition through the hydrogen evolution reaction. However, significant challenges persist in the preparation of highly loaded SACs, particularly the selection of a suitable carrier. At the same time, the search for suitable reaction conditions and operating parameters requires systematic study and optimization.

#### 4.2.5. Carbon-Based Non-Metal Catalysts

Carbon-based non-metal catalysts are regarded as promising candidates for the eNO_3_RR to NH_3_ due to their abundance, low cost, environmental friendliness, high conductivity, tunable pore structures, and modifiable surface chemistry. However, pristine carbon materials exhibit intrinsic chemical inertness, leading to weak adsorption of NO_3_^−^ and its intermediates and consequently limited catalytic activity. Doping the carbon matrix with non-metal heteroatoms such as N and fluorine (F) is an effective strategy to disrupt its inertness and create highly active sites. Heteroatom doping can induce charge redistribution and spin density polarization within the carbon skeleton, thereby modulating the adsorption behavior of reaction intermediates and significantly enhancing eNO_3_RR performance [140].

Among various dopants, N doping has been the most extensively studied. N atoms possess a higher electronegativity than carbon atoms. When incorporated into the carbon lattice, they withdraw electrons from adjacent carbon atoms, resulting in the formation of localized positive charges on the carbon sites. These positively charged carbon sites favor the adsorption and activation of anionic reactants like NO_3_^−^ [141]. The work by Du et al. [142] provides deep insight into the synergistic effect between specific nitrogen species and defects. They synthesized carbon-based catalysts (NHC-x) with controllable quaternary nitrogen groups and nitrogen vacancies through the co-assembly of hexaazatriphenylene cyanide (HAT-CN) with F127 followed by calcination at different temperatures. Among them, NHC-1000 achieved a high FE of 91.2% and an NH_3_ yield rate of 2.6 mmol h^−1^ gcat^−1^. Density functional theory (DFT) calculations revealed that the high performance originates from the synergy between quaternary-N sites and nitrogen vacancies: the quaternary-N sites effectively facilitate the potential-determining step, the protonation of NO_(ads)_ to HNO_(ads)_, while the adjacent nitrogen vacancies further cooperatively promote the formation of NH_2(ads)_ intermediates, thereby efficiently driving the conversion of NO_3_^−^ to NH_3_ and suppressing the HER.

Beyond N, other highly electronegative heteroatoms like F have also been used to tune the catalytic properties of carbon materials. F atoms, with their extremely high electronegativity and unique atomic radius, significantly perturb the sp^2^ hybridization system of carbon, introducing more unsaturated sites and defects into the carbon skeleton, which alters its electronic structure and reactivity. Li et al. [143] synthesized fluorine-doped carbon (FC) catalysts by calcining waste cigarette filters fully absorbed with a polytetrafluoroethylene (PTFE) solution. This catalyst exhibited excellent eNO_3_RR performance, with an NH_3_ yield rate as high as 23.8 mmol h^−1^ gcat^−1^, which is 2 to 4 times higher than that of undoped carbon catalysts. DFT analysis revealed that F doping not only reduces the energy barrier for the NO_3_^−^ hydrogenation step but, more importantly, effectively weakens the adsorption energy of H_(ads)_ intermediates on the carbon sites, thereby fundamentally suppressing the competing HER and improving reaction selectivity.

In summary, modulating the electronic structure of carbon-based non-metal catalysts via heteroatom doping (e.g., N, F) is an effective pathway to enhance their eNO_3_RR performance. The common mechanism lies in the dopants inducing charge redistribution within the carbon lattice due to the electronegativity difference with carbon atoms, creating active sites that optimize the adsorption energy of key intermediates (e.g., NO_(ads)_, H_(ads)_). Despite significant progress, this field still faces numerous challenges: Firstly, the intrinsic activity (e.g., turnover frequency) and electrode-area-normalized yield rate of current catalysts are generally lower than those of metal-based catalysts. Secondly, the precise structure of the active sites (e.g., the synergy between specific nitrogen configurations and defects) is difficult to characterize accurately and synthesize controllably. Furthermore, the long-term stability of dopant atoms (e.g., leaching or deactivation during electrochemical cycling) must be considered for practical applications. Future research should focus on precisely constructing highly efficient and stable active centers through strategies combining multi-heteroatom doping, defect engineering, and microstructure control, coupled with in situ characterization techniques to deeply reveal the structure-activity relationships and promote the practical application of carbon-based non-metal catalysts.

#### 4.2.6. Composite Catalysts

Composite catalysts, which integrate distinct metal and non-metal components, demonstrate pronounced advantages for eNO_3_RR by leveraging synergistic interfacial interactions. These interactions effectively regulate the electronic configurations of active sites, optimizing the adsorption energies of nitrate species and key intermediates, thereby enhancing catalytic activity and selectivity. This section focuses on emerging porous crystalline materials as composite catalyst platforms, specifically metal–organic frameworks (MOFs), covalent organic frameworks (COFs), and their hybrid derivatives, highlighting their tailored structures and interfacial engineering for advanced eNO_3_RR.

**Figure 8:**
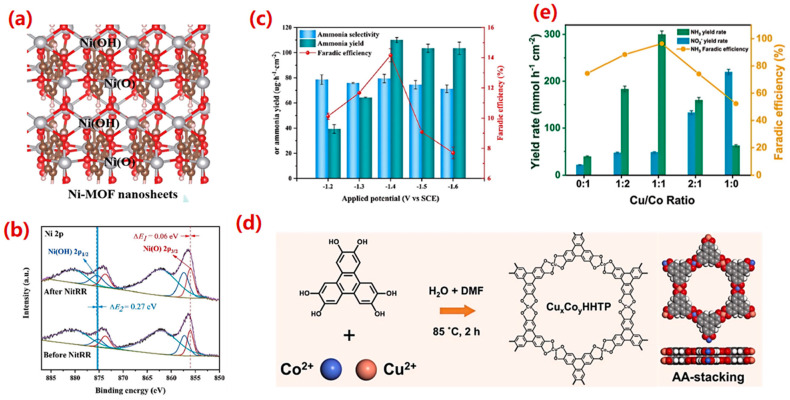
(**a**) Ni-MOF/NF catalyst structure, (**b**) Ni 2p XPS spectra of Ni-MOF/NF electrode before and after electrochemical reduction. To assure accuracy, each experiment was performed at least more than twice, and (**c**) Difference in ammonia selectivity, ammonia yield, and Faradic efficiency with Ni-MOF/NF electrode under different applied potentials [145]. Reproduced with permission 2023 Elsevier Inc. (**d**) CuxCoyHHTP cMOFs preparation process, and (**e**) NH_3_ yield rate and FE of Cu/Co catalyst [147]. Reproduced with permission 2023 Elsevier B.V.

MOFs, known for their high porosity, large surface areas, and tunable structures with uniformly distributed unsaturated metal sites, have garnered significant interest in electrocatalysis. Research strategies often involve engineering the MOFs metal nodes or loading active species to enhance performance [144]. For instance, Pan et al. [145] developed ultrathin Ni-MOF nanosheets supported on Ni foam (Ni-MOF/NF) (Figure 8a). This catalyst exhibited a large electrochemically active surface area and low charge transfer resistance. Crucially, combined experimental and theoretical analyses revealed that the coordination environment of the Ni sites dictates their activity: Ni atoms in a Ni(OH) coordination mode demonstrated superior NO_3_^−^ adsorption and catalytic activity for eNO_3_RR compared to those in a Ni(O) configuration (Figure 8b). Consequently, Ni-MOF/NF achieved an outstanding NH_3_ yield of 110.13 μg h^−1^ cm^−2^ and a high FE of 94.5% (Figure 8c).

Beyond monometallic systems, introducing a second metal center offers a powerful strategy for electronic structure modulation [146]. Liu et al. [147] designed bimetallic conductive MOFs (CuxCoyHHTP cMOFs) (Figure 8d). At an optimal Co/Cu ratio of 1:1, the Cu_1_Co_1_HHTP cMOF exhibited remarkable eNO_3_RR performance, with an NH_3_ yield rate of 299.9 μmol h^−1^cm^−2^ and an FE of 96.4% at −0.6 V vs. RHE (Figure 8e). Theoretical calculations indicated that the adjacent Co sites electronically perturb the Cu sites, lowering the free energy change (ΔG) of the potential-determining step in the reaction pathway.

COFs, characterized by robust covalent bonds, long-range order, and permanent porosity, provide another platform for constructing well-defined catalytic sites [148,149]. Lv et al. [150] synthesized a 2D nickel porphyrin-based COF (NiPr-TPA-COF) tailored for eNO_3_RR. This material, featuring a highly ordered structure with square nanopores, achieved a high NH_3_ yield rate of 2.5 mg h^−1^ cm^−2^ and a high FE of 90% under mild overpotentials, demonstrating excellent stability under pulse electrolysis conditions.

To circumvent the stability issues associated with the labile metal-linkage bonds in traditional MOFs under applied potentials, hybrid metal-covalent organic frameworks (c-MCOFs) have been developed by integrating covalent linkages [151]. Huang et al. [152] fabricated M-HATN-COFs with a high density of monometallic sites (M ≈ 12.5 at%) via a one-pot coordination-condensation strategy. These frameworks serve as ideal platforms for mechanistic studies. Electrocatalysts featuring both Mo and Ni monometallic sites within this structure exhibited an impressive NH_3_ yield rate of 8.52 mg h^−1^ cm^−2^, an FE of 91.3%, and remarkable stability.

Above all, composite electrocatalysts are designed through the rational integration and coupling of multiple components (e.g., MOFs, and COFs), aiming to combine the advantages of individual constituents and achieve functional synergy and performance enhancement. Such catalysts demonstrate great potential in improving nitrogen reduction selectivity and stability. However, their construction typically involves multi-step synthesis processes, making fine control of heterogeneous interfaces highly challenging. The structural complexity and inhomogeneity of composite materials also complicate the precise elucidation of active centers and reaction mechanisms. Furthermore, scalable and controllable preparation techniques still require further development.

### 4.3. Catalyst Design Strategies

The common strategies in eNO_3_RR can be categorized into two interconnected themes: (i) Electronic Structure Modulation, which directly tunes the intrinsic activity of active sites through atomic-scale engineering (e.g., bimetallic/alloying, defect/doping), and (ii) Surface and Interface Engineering, which optimizes the geometric arrangement of active sites and reaction environment (e.g., facet control, morphology control). These strategies often work synergistically to enhance catalytic performance [27].

#### 4.3.1. Bimetallic or Alloy Strategy

The bimetallic or alloy strategy enables fundamental electronic modulation, primarily by shifting the d-band center of the active metal via ligand (electronic) and strain (geometric) effects. This fine-tuning allows for the optimization of adsorption strengths for critical intermediates, thereby lowering the energy barriers of potential rate-determining steps. A sophisticated extension of this approach is the design of tandem catalytic systems, where distinct metallic sites are spatially organized to preferentially catalyze sequential steps in eNO_3_RR. For example, Jang et al. [153] masterfully illustrated this concept in a bimetallic covalent organic framework featuring atomically dispersed Cu and Co sites. In this engineered tandem system, the electronic structure of Cu sites was optimized for a strong affinity toward NO_3_^−^, driving its efficient reduction to NO_2_^−^. Concurrently, adjacent Co sites, tailored for an optimal hydrogen binding energy, facilitated the subsequent critical six-electron reduction of NO_2_^−^ to NH_3_. This spatial and functional decoupling prevented nitrite accumulation and synergistically enhanced the overall Faradaic efficiency and yield.

#### 4.3.2. Synergistic Effect of Defect Engineering and Doping

Defect engineering (e.g., creating vacancies) and heteroatom doping serve as powerful, complementary techniques for atomic-scale electronic manipulation. Defects generate coordinatively unsaturated sites with altered local charge density, while doping introduces foreign atoms that directly perturb the electronic structure of the host. Their synergistic application can create unique active centers unattainable by either method alone, often leading to superior performance by simultaneously enhancing NO_3_^−^ activation and suppressing HER [154,155,156]. For instance, Ge et al. [157] have proposed a strategy to boost defect generation through S-doping induced NiFe-LDH lattice distortion, and successfully optimized the balance of H_(ads)_ production and binding. In situ characterization and DFT calculations showed that the sulfur-mediated defect leads to the d-band center displacement of Ni and Fe sites, which efficiently promotes the enrichment of NO_3_^−^ and inhibits the binding of H_(ads)_. By rationally regulating the type and concentration of defects as well as the type of dopant atoms, precise optimization of the catalyst’s electronic structure and surface properties can be achieved, thereby comprehensively enhancing its catalytic activity, selectivity, and stability.

The primary challenge lies in the precise control over the type, density, and spatial distribution of defects and dopants. Furthermore, the stability of these engineered sites under prolonged electrochemical operation remains a critical concern. Future directions involve developing synthetic methods for precise defect/dopant control and understanding the dynamic behavior of these sites under working conditions.

#### 4.3.3. Facet and Morphology Control

Facet engineering is the process of regulating the exposed crystal planes of a catalyst, altering its surface structure and electronic properties, optimizing the distribution of active sites and reaction pathways, thereby significantly enhancing catalytic performance. For instance, Zhong et al. [158] have modulated the surface oxygen species of Cu_2_O via facet engineering, and studied the impact of these oxygen species on the eNO_3_RR activity. They have found that while oxygen vacancies on Cu_2_O(111) surface promote the adsorption of reactants and reaction intermediates, hydroxyl groups effectively inhibit the side reaction of hydrogen evolution and facilitate the hydrogenation process of eNO_3_RR. These two effects work in concert to render Cu_2_O(111) facet the highest eNO_3_RR activity relative to those from other facets.

Morphology control, by regulating the structure and surface characteristics of the catalyst, can effectively enhance the efficiency of mass transfer and charge transfer, thereby improving the NH_3_ yield and FE of eNO_3_RR. Exemplary Case, such as Zhu et al. [159] have provided a viable strategy to enhance mass transfer at the catalytic interface through rational morphology control, boosting the intrinsic activity of catalysts in the NO_3_RR process. They incorporated a Cu-bipyridine catalytic interface and fabricated crystalline 2D covalent organic framework films with significantly exposed catalytic sites, leading to improved FE and NH_3_ yield compared to bulk catalysts.

A significant challenge is the stabilization of high-energy facets and the scalable, reproducible synthesis of catalysts with complex, well-defined morphologies. Additionally, catalysts may undergo reconstruction under reaction conditions, altering their initial facet and morphology. Future work should focus on stabilizing desired structures and employing operando microscopy to monitor these changes.

## 5. Conclusions and Outlook

The eNO_3_RR presents a strategic pathway for sustainable nitrogen management by integrating environmental remediation with the production of valuable chemicals. Despite considerable progress in mechanistic understanding and catalyst development, significant challenges impede its commercial applications. To advance the sustainable development of eNO_3_RR, we herein identify key bottlenecks and propose innovative solutions across several critical areas:

Mechanistic elucidation: While the general reaction pathways for eNO_3_RR are largely established, a deeper understanding of the dynamic evolution of key intermediates (e.g., NO_(ads)_, N_(ads)_, NH_2(ads)_) and their interactions with catalytic active sites is crucial. This knowledge gap currently hinders the rational design of catalysts with high NH_3_ selectivity. Future work must leverage advanced in situ/operando characterization techniques, such as X-ray absorption fine structure (XAFS), Raman spectroscopy, and electrochemical mass spectrometry, to monitor the chemical state and structural evolution of active centers in real time, thereby establishing dynamic structure-activity relationships.

Rational catalyst design: Although many catalysts demonstrate excellent performance under benign laboratory conditions (e.g., low current density, pure electrolytes), their activity, selectivity, and stability are severely challenged under industrially relevant conditions, such as high current densities, long-term operation, and complex real wastewater matrices. Future research should target industrial-performance benchmarks, including a current density > 500 mA cm^−2^, FE > 90%, operational stability > 1000 h, and energy consumption < 40 kW h kg^−1^ NH_3_. Achieving these goals will require the development of novel catalysts via green and scalable synthesis routes to ensure high activity, selectivity, and durability.

System-level engineering and optimization: The development of advanced electrochemical reactors is essential for stable, high-current-density NH_3_ production. Exploring multi-field coupling strategies (e.g., microwave, ultrasound, plasma) could help overcome kinetic limitations, while process intensification through advanced separation technologies (e.g., membrane separation, temperature-modulated concentration swing) can enhance both NH_3_ recovery efficiency and product purity. Furthermore, direct integration with renewable energy sources (e.g., solar, wind) is critical for achieving truly carbon-neutral NH_3_ synthesis.

Assessment under real-world conditions: A comprehensive techno-economic analysis (e.g., incorporating material, energy, and separation costs) is imperative to evaluate the feasibility of large-scale implementation. Such an assessment should be coupled with validation using real industrial wastewater to bridge the gap between idealized laboratory studies and practical application scenarios.

In summary, eNO_3_RR technology holds dual promise for mitigating NO_3_^−^ pollution and enabling green NH_3_ synthesis. Continued breakthroughs in mechanistic understanding, catalyst design, and reactor engineering are essential to unlock its full potential for environmental sustainability and the future of chemical production.

## Data Availability

No new data were created or analyzed in this study. Data sharing is not applicable.
